# Religious Affiliation and Marital Satisfaction: Commonalities Among Christians, Muslims, and Atheists

**DOI:** 10.3389/fpsyg.2019.02798

**Published:** 2019-12-13

**Authors:** Piotr Sorokowski, Marta Kowal, Agnieszka Sorokowska

**Affiliations:** Institute of Psychology, University of Wrocław, Wrocław, Poland

**Keywords:** religious affiliation, marital satisfaction, Christians, Muslims, atheists

## Abstract

Scientists have long been interested in the relationship between religion and numerous aspects of people’s lives, such as marriage. This is because religion may differently influence one’s level of happiness. Some studies have suggested that Christians have greater marital satisfaction, while others have found evidence that Muslims are more satisfied. Additionally, less-religious people have shown the least marital satisfaction. In the present study, we examined marital satisfaction among both sexes, and among Muslims, Christians, and atheists, using a large, cross-cultural sample from the dataset in Sorokowski et al. (2017). Our results show that men have higher marital satisfaction ratings than women, and that levels of satisfaction do not differ notably among Muslims, Christians, and atheists. We discuss our findings in the context of previous research on the association between marriage and religion.

## Introduction

Religion has been present nearly since the dawn of human history (Dickson, 1992; Clottes, 2006). Nowadays, it continues to play a key role in most societies (Tarakeshwar et al., 2003). Almost 82% of the world’s people affiliate themselves with a religion. By country, only 5% of Americans, 4% of South Africans, 1% of Brazilians, and 3% of Indians claim to be atheists (Win-Gallup International Poll, 2012). It therefore seems viable that we should deepen our understanding on how religion influences our lives (Miller and Thoresen, 2003; McCleary and Barro, 2006). As religion is strongly connected with a wide range of relationship-related values and norms (Zarean and Barzegar, 2016), the link between religion and family life has been among the most widely discussed topics in the social sciences (Thomas and Cornwall, 1990; Ammerman and Roof, 1995; Christiano, 2000). The present study aimed to investigate one of those aspects: the association between religion and marital satisfaction.

A large number of studies have addressed marriage in the context of religion (including topics such as marital stability: Heaton and Albrecht, 1991; Lehrer and Chiswick, 1993; Call and Heaton, 1997; Orathinkal and Vansteenwegen, 2006; Brown et al., 2008; Mahoney et al., 2008; Vaaler et al., 2009; marital problem solving: Hünler and Gençöz, 2005; marital fidelity: Dollahite and Lambert, 2007; and marital dependency: Wilson and Musick, 1996). Nevertheless, few data exist on how religious affiliation affects marital satisfaction; and even when studies have taken up this topic, they have had limitations. For example, they have mainly focused on various dimensions of religiosity (e.g., church attendance, religious homogamy, and theological conservatism), and not the specific religion (Schumm et al., 1989; Shehan et al., 1990; Booth et al., 1995; Brandt, 2004; Gaunt, 2006; Vaaler et al., 2009; Wade and Wiloso, 2016); examined people of different religions, but without further comparing the effects of each religion on marital satisfaction (Sullivan, 2001; Williams and Lawler, 2003; Olson et al., 2016); looked at members of one religion (Christian: Shehan et al., 1990; Anthony, 1993; Booth et al., 1995; Sullivan, 2001; Williams and Lawler, 2003; Asamarai et al., 2008; Lichter and Carmalt, 2009; Christian and Jewish: Heaton, 1984(Muslim: Al-Othman, 2012; Fard et al., 2013; Al-Darmaki et al., 2016; Mormon: Schramm et al., 2012); been conducted in one country (United States: Brandt, 2004; Marks, 2005; Brown et al., 2008; Schramm et al., 2012; Israel: Gaunt, 2006; Iran: Fard et al., 2013 (United Arab Emirates: Al-Othman, 2012; Al-Darmaki et al., 2016; Ghana: Dabone, 2012); or included only low-income married couples (Lichter and Carmalt, 2009).

Furthermore, results from those studies brought mixed results. Some research has suggested that religious couples are happier with their marriages than are non-religious couples (Ortega et al., 1988; Anthony, 1993; Brandt, 2004; Mahoney et al., 2008; Schramm et al., 2012), with Christians showing greater happiness than Muslims (Lev-Wiesel and Al-Krenawi, 1999, but for contradictory results see Abu-Rayya, 2007), while other studies have provided evidence of a weak association between marital satisfaction and religion (Booth et al., 1995; Sullivan, 2001; Gaunt, 2006; Orathinkal and Vansteenwegen, 2006; Al-Othman, 2012; Fard et al., 2013), and a few indicated there is no link (Williams and Lawler, 2003; Luo and Klohnen, 2005; Dabone, 2012; Olson et al., 2016).

There is even less evidence of the relationship between atheism and marital satisfaction. Some studies have shown a positive association between religiosity and marital satisfaction (Lichter and Carmalt, 2009; Wilcox and Wolfinger, 2008), or mental well-being (Galen and Kloet, 2011), which would suggest atheists (at the low end of the religiosity continuum) may have lower indicators of marital happiness than religious adherents. Nevertheless, to date, researchers have not directly addressed the issue of atheists in the marriage context, as existing studies have generally focused on religious married couples (Giblin, 1997; Fincham et al., 2011), thus excluding the relatively large number of atheists. These prevent drawing of certain conclusions about marital satisfaction among non-religious people.

In sum, no cross-cultural studies have compared marital satisfaction among people of various religious affiliations (or non-religious: atheists). One can only hypothesize why scientists are not willing to tackle this issue. One reason might be that such comparisons are often perceived as politically incorrect (Raines, 1996). Nevertheless, comparisons within a single religion or culture do not permit generalization of the conclusions, as people of different religious affiliations might be subject to different, culture-specific pressures and situations (Tarakeshwar et al., 2003). To fill this gap in knowledge, the present study aimed to analyze the relationship between religious affiliation and marital satisfaction, in a large, cross-cultural sample.

## Materials and Methods

### Participants

We used data from the dataset published in Sorokowski et al. (2017), a study conducted between July 2012 and December 2013. Participants, after providing informed consent, completed a written questionnaire (except in two countries where data were collected online), and were not compensated for their participation (except in one country where participants received 50 Hong Kong dollars). See Sorokowski et al. (2017) for more details regarding the participants in the dataset.

The present study’s main goal was to compare people from the world’s most common religions (Grim et al., 2015). The Sorokowski et al. (2017) dataset included Protestants, Christians, Jews, Muslims, Buddhists, Atheists, Jehovah’s Witnesses, Evangelicals, Spiritualists, Orthodox, Hindus, and others not of the aforementioned religious affiliations. Despite this wide representation, we decided to only analyze answers provided by Christians, Muslims, and atheists, as these were the most highly represented religious and non-religious samples deriving from the different countries in the dataset (Christians from 27 countries; Muslims from 23 countries, and atheists from 27 countries; see Tables 1, 2). Thus, in contrast with studies of people from a single country (e.g., Brown et al., 2008; Vaaler et al., 2009), our study aimed to compare religious affiliation and marital satisfaction among various nationalities.

**TABLE 1:** Characteristics of study population’s religious affiliation and sex distribution from each country.

**Country**	**Total number of participants**	**Religion**								
		
		**Christians**			**Muslims**			**Atheists**		
				
		**Men**	**Women**	**Total**	**Men**	**Women**	**Total**	**Men**	**Women**	**Total**
Brazil	256	90	46	136	8	13	21	61	38	99
Bulgaria	1	0	0	0	1	0	1	0	0	0
Canada	55	6	7	13	2	1	3	12	27	39
China	106	0	1	1	0	2	2	43	60	103
Croatia	585	224	250	474	4	1	5	62	44	106
Estonia	97	0	4	4	0	0	0	36	57	93
Germany	69	6	8	14	0	1	1	20	34	54
Ghana	35	13	12	25	3	3	6	1	3	4
Greece	9	0	0	0	0	0	0	5	4	9
Hong Kong	65	11	4	15	0	0	0	31	19	50
Hungary	225	48	116	164	0	0	0	22	39	61
India	33	3	1	4	7	12	19	5	5	10
Indonesia	4	0	0	0	0	3	3	1	0	1
Iran	587	0	0	0	258	329	587	0	0	0
Italy	306	94	181	275	0	0	0	24	7	31
Kazakhstan	70	10	10	20	28	22	50	0	0	0
Kenia	43	16	16	32	4	6	10	1	0	1
Malaysia	87	1	0	1	40	46	86	0	0	0
Mexico	138	59	63	122	1	1	2	6	8	14
Nigeria	301	119	100	219	14	38	52	13	17	30
Pakistan	130	0	0	0	59	71	130	0	0	0
Poland	434	152	256	408	0	0	0	13	13	26
Portugal	271	84	160	244	0	2	2	15	10	25
Romania	6	1	3	4	0	0	0	0	2	2
Russia	31	0	2	2	0	0	0	22	7	29
Saudi Arabia	198	0	0	0	86	112	198	0	0	0
Slovakia	216	39	112	151	0	0	0	30	35	65
South Korea	33	3	1	4	0	0	0	18	11	29
Spain	197	61	73	134	1	0	1	29	33	62
Switzerland	107	42	18	60	0	2	2	26	19	45
Turkey	390	1	0	1	228	146	374	10	5	15
United Kingdom	61	8	14	22	2	1	3	20	16	36
Uganda	50	22	11	33	6	9	15	1	1	2

**TABLE 2:** Characteristics of studied Christians, Muslims, and atheists, and their marital satisfaction.

**Religion**	**Number of countries**	**Number of participants**	**Marital satisfaction**
Christian	27	2582	5.69(SD = 1.42)
Muslim	23	1572	5.72(SD = 1.42)
Atheistic	27	1041	5.69(SD = 1.43)

In total, analysis included answers of 5,195 participants [mean age: 41.35; standard deviation (SD): 11.54; range: 18–88 years], among whom 2,393 (46.06%) were men.

### Procedure

All questionnaires were translated into the participants’ local language (with the exception of English-speaking countries, where questionnaires were administered in their original wording), using back-translation (Brislin, 1970). Participants were surveyed (*inter alia*) via two questionnaires that inquired on marital satisfaction. In the present study, one questionnaire, the Kansas Marital Satisfaction Scale (KMSS) (Nichols et al., 1983; Schumm et al., 1986), was included in the analysis. The KMSS is a well-established and commonly used tool for assessing satisfactory psychometric characteristics (Schumm et al., 1986; Crane et al., 2000), and was successfully used in studies involving non-Western samples (Shek and Tsang, 1993). Sorokowski et al. (2017) found the scale was culturally equivalent (Tucker’s phi coefficient from 0.92 to 1), and reliable (Cronbach’s alpha on the pooled data reached 0.94). Questions from the scale include: “How satisfied are you with your marriage?” and “How satisfied are you with your wife/husband as a spouse?” Participants answered each item on a seven-point scale ranging from 1 (extremely dissatisfied) to 7 (extremely satisfied). In all subsequent analysis, as a measure of marital satisfaction we used mean scores of each participant’s KMSS answers.

Participants were asked directly about their religious affiliation. Certain variables that in previous studies have been shown to correlate with marital satisfaction—such as age, length of relationship, education, number of children, and material situation (Bradbury et al., 2000; Twenge et al., 2003; Sorokowski et al., 2017)—were also included in the analysis. Detailed information about the procedure for data collection can be found in Sorokowski et al. (2017).

## Results

**FIGURE 1:**
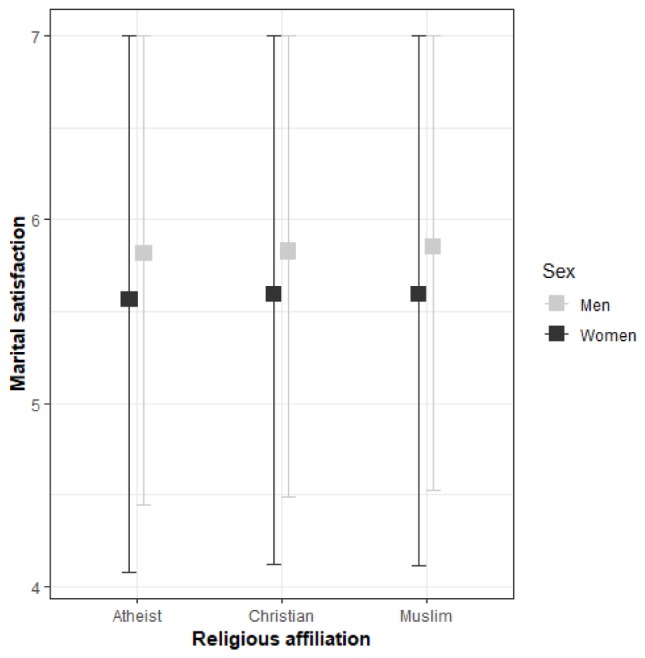
Men’s and women’s marital satisfaction among the studied Christians, Muslims, and atheists.

We conducted an analysis of covariance to determine the relation between religious affiliation and marital satisfaction. There was a non-significant effect of the former on the latter, after controlling for sex, age, length of marriage, number of children, education, and material situation [*F*(2,5194) = 1.11, *p* = 0.33]. Analysis showed that some variables were significant covariates, including: age [*F*(1,5194) = 6.99, *p* < 0.05]; material status [*F*(1,5194) = 98.34, *p* < 0.001]; and sex [*F*(1,5194) = 34.28, *p* < 0.001]. Overall, men (mean = 5.83, SD = 1.34) had higher marital satisfaction than women (mean = 5.59, SD = 1.48) (see Figure 1). Nevertheless, this effect was extremely weak (*Eta* < 0.01).

## Discussion

The present study’s primary goal was to examine the association between religious affiliation and marital satisfaction, and the results showed that there was no relationship between the former and level of the latter—Christians and Muslims were found to be similarly satisfied with their marriages, as were atheists. Nevertheless, the present analysis provided support for a link between marital satisfaction and age (younger people showed higher marital happiness), material status (higher material status, higher marital satisfaction), or sex (men were happier in their marriages than women).

Previous findings have indicated Abrahamic religions (e.g., Christianity, Islam) share many similarities (Agius and Chircop, 1998; Zarean and Barzegar, 2016) and promote formation of traditional family ties, such as marriage rather than cohabitation, and marital rather than non-marital births (Dollahite and Lambert, 2007; Zarean and Barzegar, 2016). However, these religions have some substantive differences in beliefs and practices. For example, polygyny is not accepted in Christianity, whereas it is widely accepted in Islam, and such a family model may negatively influence marital life (Al-Krenawi and Graham, 2006). Despite the discrepancies between those two religions, the present study found no differences between them as far as marital satisfaction, and this included people from different parts of the world.

Moreover, since the New York City terrorist attacks on September 11, 2001, Islam has been central in many debates, discussions, and publications (Alghafli et al., 2014). Discussion on Islam frequently concerns familial issues, perceived by the Western media mostly in a negative light. Problematic issues include, for instance, gender roles and the treatment of women (McDonald, 2006; Ridouani, 2011; Ennaji, 2016). Studies, however, do not support this unfavorable view of females’ situations: religious Muslims show increased marital satisfaction (Abdel-Khalek, 2006, 2010; Asamarai et al., 2008; Ahmadi and Hossein-Abadi, 2009; Zaheri et al., 2016, but see also Abu-Rayya, 2007).

The present study’s results provide evidence that Christians and Muslims do not differ in their level of marital satisfaction. People from various countries identifying themselves as belonging to one of these two religions had similar level of marital happiness, which is consistent with previous findings. For instance, Dabone (2012) compared marital satisfaction among Muslim and Christian spouses, and found relative dissatisfaction, while the religious affiliation did not affect the satisfaction.

As scarce data exist on marital satisfaction among atheists, the present study’s second aim was to investigate whether atheists have similar marital satisfaction to marriages as do religious adherents. Considering positive correlations found between religiosity and marital satisfaction (Marks, 2005), atheists may be expected to have significantly lower levels of both variables. A major drawback of previous related research is its predominant focus on comparisons between more-religious and less-religious people (Fincham et al., 2011), excluding the relatively large group that atheists represent. Additionally, most studies have been conducted in the United States, where atheists are often negatively stereotyped (Zuckerman, 2009). The present study results provide evidence that atheists are neither more nor less satisfied with their marriages than religious adherents, which suggests religion may not influence marital satisfaction.

There are a few possible explanations for observed similar marital satisfaction ratings across people of different religions. Overall, married couples constitute a lower percentage of people in a relationship (Nock, 1995). Those who decide to get married may be particularly committed or well-suited to partnership, regardless of their religious affiliation. Entering a serious relationship, such as marriage, requires strong enthusiasm toward the partner (Wang and Chang, 2002) and, thus, results in higher ratings of subjectively perceived relationship satisfaction. Another possible explanation may be that people generally consider marriage a long-lasting relationship (Silliman and Schumm, 2004; Willoughby and Dworkin, 2009), and when they decide to get married, they rationalize and “cognitively close” their choice (Webster and Kruglanski, 1994). Participants in the study population may have felt they had to be satisfied with their relationship, as they had invested so much energy into its development. Had they reported being unsatisfied, feeling an internal conflict may have surfaced (e.g., “Why am I even with him/her if it makes me unhappy?”). The need to explain the dissonance of staying in an unsuccessful relationship would be negatively perceived, and could yield unpleasant emotions, especially in Western, individualistic cultures, which value the pursuit of personal happiness at all costs (Gilovich et al., 2015). Such emotion could also occur in Eastern, collectivistic cultures, which emphasize the importance of being unselfish, grateful, and appreciative of one’s partner (Kagawa-Fox, 2010).

In general, participants were relatively satisfied with their marriages. Nonetheless, men’s marital satisfaction differed from women’s (independent of religious affiliation). Over 40 years ago, Bernard (1975) presented a provocative and controversial thesis asserting marriage is better for men than for women, and his statement has raised heated discussions. Most of the research has provided evidence for to support Bernard’s (1975) that thesis (Fowers, 1991; Schumm et al., 1998), and this is also true in non-Western cultures (Shek and Tsang, 1993; Asamarai et al., 2008). However, there was also one study which yielded unclear findings (McNulty et al., 2008). Results of the present study – which is based on the analysis of a large, cross-cultural sample, confirm the differences among men’s and women’s marital satisfaction: husbands did indeed have higher marital satisfaction than wives. Nevertheless, the size effect of these sex differences was extremely small (*Eta* < 0.01).

In conclusion, despite a large body of research on marital satisfaction (Bradbury et al., 2000; Twenge et al., 2003; Hilpert et al., 2016), most studies have rarely controlled for participants’ religion. Even when they have done so, they have not explored the differences between people of various religious affiliations (Sullivan, 2001; Williams and Lawler, 2003; Olson et al., 2016). Future research should therefore focus on people of different (1) religions (especially less-prevalent ones); and (2) cultures (as most studies up to date have been conducted on Western, educated, industrialized, rich, and democratic populations (Henrich et al., 2010), and should take into consideration other factors that may influence marital satisfaction among people of different religious affiliations (e.g., number of children, education, country’s development), as this would provide further understanding on the interaction between religion and marital happiness, as well as culture.

## Data Availability Statement

Publicly available datasets were analyzed in this study. This data can be found here: https://figshare.com/s/d2bd33a9605a3a204881.

## Ethics Statement

The studies involving human participants were reviewed and approved by the Institutional Review Board of the Institute of Psychology, University of Wrocław. The patients/participants provided their written informed consent to participate in this study.

## Author Contributions

All authors contributed in conducting the statistical analysis and preparing the manuscript.

## Conflict of Interest

The authors declare that the research was conducted in the absence of any commercial or financial relationships that could be construed as a potential conflict of interest.
